# Development of CliniPup, a Serious Game Aimed at Reducing Perioperative Anxiety and Pain in Children: Mixed Methods Study

**DOI:** 10.2196/12429

**Published:** 2019-06-01

**Authors:** Sarah Verschueren, June van Aalst, Anne-Marie Bangels, Jaan Toelen, Karel Allegaert, Connor Buffel, Geert Vander Stichele

**Affiliations:** 1 MindBytes BVBA Merksplas Belgium; 2 Division of Nuclear Medicine and Molecular Imaging Department of Imaging and Pathology KU Leuven Leuven Belgium; 3 Department of Development and Regeneration KU Leuven Leuven Belgium; 4 Division of Neonatology Department of Pediatrics Erasmus MC-Sophia Children’s Hospital Rotterdam Netherlands; 5 MindLab Interactive AI Inc Edmonton, AB Canada

**Keywords:** serious games for health, behavior change, perioperative pain, perioperative anxiety, pediatric, ambulatory surgery

## Abstract

**Background:**

An increasing number of children undergo ambulatory surgery each year, and a significant proportion experience substantial preoperative anxiety and postoperative pain. The management of perioperative anxiety and pain remains challenging in children and is inadequate, which negatively impacts the physical, psychosocial, and economic outcomes. Existing nonpharmacological interventions are costly, time consuming, vary in availability, and lack benefits. Therefore, there is a need for an evidence-based, accessible, nonpharmacological intervention as an adjunct to existing pharmacological alternatives to reduce perioperative anxiety and pain in children undergoing ambulatory surgery. Technology-enabled interventions have been proposed as a method to address the unmet need in this setting. In particular, serious games hold a unique potential to change health beliefs and behaviors in children.

**Objective:**

The objective of this research was to describe the rationale, scientific evidence, design aspects, and features of CliniPup, a serious game aimed at reducing perioperative anxiety and pain in children undergoing ambulatory surgery.

**Methods:**

The SERES Framework for serious game development was used to create the serious game, CliniPup. In particular, we used a mixed methods approach that consisted of a structured literature review supplemented with ethnographic research, such as expert interviews and a time-motion exercise. The resulting scientific evidence base was leveraged to ensure that the resulting serious game was relevant, realistic, and theory driven. A participatory design approach was applied, wherein clinical experts qualitatively reviewed several versions of the serious game, and an iterative creative process was used to integrate the applicable feedback.

**Results:**

CliniPup, a serious game, was developed to incorporate a scientific evidence base from a structured literature review, realistic content collected during ethnographic research such as expert interviews, explicit pedagogical objectives from scientific literature, and game mechanics and user interface design that address key aspects of the evidence.

**Conclusions:**

This report details the systematic development of CliniPup, a serious game aimed at reducing perioperative anxiety and pain in children undergoing ambulatory surgery. Clinical experts validated CliniPup’s underlying scientific evidence base and design foundations, suggesting that it was well designed for preliminary evaluation in the target population. An evaluation plan is proposed and briefly described.

## Introduction

### Background

According to the latest data, over 25,000 ambulatory surgical procedures are performed on children aged 6-10 years in Belgium each year [1]. Moreover, ambulatory surgical interventions in children are increasing in developed nations such as the United States and United Kingdom [2-3]. Ambulatory surgical procedures are associated with significant levels of perioperative pain and anxiety in children, with 40%-60% experiencing high levels of anxiety on the day of surgery and >30% experiencing moderate-to-severe postoperative pain [4-6]. Inadequate management of perioperative pain and anxiety results in negative short- and long-term physical and psychosocial outcomes such as delirium, sleep disturbances [7-10], delayed wound healing, postoperative immunosuppression, increased susceptibility to infection, and distrust of health care practitioners (HCPs), which is a risk factor for future health care avoidance [5,11-13]. As a bidirectional relationship between pain and anxiety has clearly been established, perioperative anxiety can influence a child’s pain experience and vice versa [14-16]. Inadequate management of perioperative pain and anxiety also results in negative economic outcomes such as HCP burden, child absenteeism, parent absenteeism, inpatient admission, and increased analgesic and anxiolytic consumption [5,11-13,17]. In addition, preoperative anxiety in both children and their parents affects children’s experience of perioperative pain [7-9,16].

Management of perioperative pain and anxiety is particularly challenging in children, because they experience pain and anxiety differently from adults, have limited skills to communicate pain and anxiety, and often have a limited understanding of the surgery purpose and process [8,18-20]. Furthermore, the use of pharmacological interventions to alleviate preoperative anxiety, such as anxiolytics, is associated with adverse effects (eg, paradoxical reactions) and, in certain cases, delay discharge [11,12,21,22]. Adverse effects also pose a problem for pharmacological treatments targeted at perioperative pain and as such, HCPs and parents may inadequately manage postoperative pain, leading to undertreatment [23-30].

Although there is minimal standardization across surgical centers, nonpharmacological interventions are also used to address perioperative anxiety and pain in children. These interventions vary in their objectives, with some offering distraction or information only and others focusing on behavior change (coping strategies, communication skills, etc). Nonetheless, the majority remain costly, time-consuming, and surgical-center specific [8,9,19,31,32]. In contrast, other interventions such as distractions may also be effective in reducing preoperative anxiety [9,31,33]. Although these options are less constrained by cost and time compared to the alternatives, they transiently reduce anxiety [31], offering little in the way of information provision, modeling, and coping skills—the three most effective strategies to prepare children for surgery [8]. This is particularly relevant in children aged >4 years who prefer to maintain control [9,19].

One potential nonpharmacological intervention that has shown efficacy in children is serious games. These digital tools can not only be inexpensively and widely deployed, but also offer the potential to transfer knowledge and educate users in an interactive and engaging manner, which can lead to behavior change [34-36]. Aligned with these features, serious games have proven to be particularly useful when targeted toward children in health care settings [37-39]. In fact, there are a number of studies investigating digital interventions aimed at reducing child perioperative pain and anxiety [33,40-42]. However, most of these options rely on pure distraction. The approach explored in this research focuses on achieving behavior change by educating children in a fun and engaging manner through experiential learning. The use of experiential learning is aligned with the theory of Cognitive Neuroeconomics, which states that individuals revert to experiential thinking, as opposed to rationale thinking, when under stress [43]. In the context of perioperative pain and anxiety, this suggests that children need *information* on various aspects of surgery and behavioral skills (communication and coping techniques) and to *experience* their surgical journeys (purpose, process, etc) in a safe and playful environment [8,19]. This thinking is also aligned with the optimal strategies to prepare children for surgery (information, modeling, and coping skills), which has been described by Fortier et al [42] and is consistent with various psychological and behavior change models [43-46].

### Objective

The objective of this research was to develop an evidence-based serious game aimed at reducing perioperative anxiety and pain in children undergoing ambulatory surgery. The purpose of this paper was to describe the rationale, scientific evidence, and subsequent translation of that framework into design and game mechanic features that comprise the serious game CliniPup.

## Methods

### Overview

**Figure 1:**
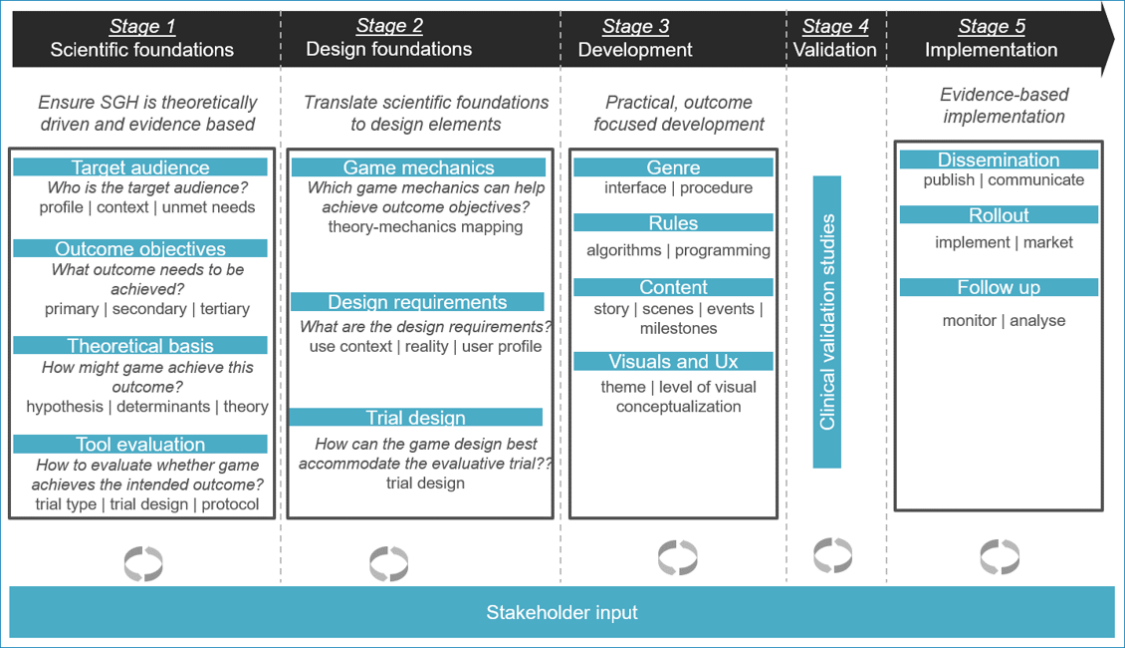
SERES Framework used to develop CliniPup. Adapted from Verschueren et al [47].

To create an evidence-based game that is purposefully designed to address relevant challenges faced by the target end user and change behavior, CliniPup was developed using the SERES Framework [47]. The SERES Framework, developed by the authors SV, CB, and GVS, was applied to ensure that the serious game was theory driven and evidence based [47]. The framework covers all aspects of the development process (scientific, technological, and design) and is transparently described in sufficient detail to allow developers to implement it in a wide variety of projects, irrespective of discipline, health care segment, or focus. It consists of five distinct stages (Figure 1). Each stage has a specific focus and is informed by various stakeholders. Several iterations of development may occur within a given stage, progressively refining the serious game based on testing and feedback from relevant stakeholders [47]. The application of this method in the context of pediatric perioperative anxiety and pain is described in the sections below, with a focus on Stages 1, 2, and 3, which represent the underlying Scientific Foundations, Design Foundations, and Development, respectively.

### Scientific Foundations

#### Target Audience

Sound Scientific Foundations for CliniPup were established at the earliest stage of development to ensure that the game was relevant, theoretically driven, and evidence based, in line with the governing research methodologies. This stage assessed, based on objective criteria, whether there was a relevant unmet medical need for a clearly defined target audience that can be addressed with a serious game. In line with this aim, an exploratory review of the research literature on perioperative anxiety and pain was performed. To this end, the PubMed database was search electronically from March to April 2015 with a search strategy (terms used in duplicate: pain, anxiety, surgery, perioperative, children, and pediatric). The following criteria were applied for inclusion: English language and review. Similarly, the following criteria were applied for exclusion: published before 2000 and nonpediatric population.

#### Outcome Objectives

This stage assessed what the objectives of a serious game should be in the context of the target audience defined in the first step. Evidence collected in the exploratory literature search was reviewed again with the aim of defining the outcome objectives.

#### Theoretical Basis

Formulating a hypothesis of how a serious game might achieve the intended outcome objectives is a vital step toward purposeful design and the evaluation and validation of its causal effect on the outcome [47]. In particular, modifiable barriers and drivers (outcome determinants) contributing to pain, anxiety, and control in children were collected from the scientific literature. To this end, a critical review of the scientific literature was performed. From September to October 2015, the PubMed database was searched electronically with the following search strategy: pain OR children OR preoperative, postoperative OR anxiety OR induction OR perioperative OR education OR pharmacological treatment OR non-pharmacological treatment OR determinants. The following criteria were applied for inclusion: English language, review, case study, retrospective study, and clinical trial. The criteria for exclusion were published before 2000, nonpediatric population, and pharmacological intervention only.

Barriers and drivers (determinants) were extracted directly from the literature and classified into categories, thereby arriving at a manageable amount of key factors. The relative importance of each determinant was also defined based on qualitative descriptions in the selected articles. Moreover, various experts in the field such as nurses, pediatricians, and surgeons were consulted to evaluate the resulting determinant categories.

Consistent with the SERES Framework Methodology, learning objectives were defined for each determinant and subsequently mapped within a theoretical framework. The Information-Motivation-Behavioral skills (IMB) model of behavior change was selected as the theoretical framework because it has been used in the design of health promotion interventions and was aligned with educational strategies to prepare children for surgery in the literature [8,44]. These learning objectives, in turn, guided the Design Foundations.

It also became evident that there were a substantial number of clinical practice documents available, which were not considered primary research. Therefore, the secondary literature on educational programs for children in the perioperative setting was also reviewed. These findings played an important role in informing the learning objectives and various game design aspects, which are described in the Results section.

#### Tool Evaluation

Both the literature collected via the exploratory search and the critical review were analyzed to understand how to validate a serious game in the context of a nonpharmacological intervention aimed at addressing perioperative anxiety and pain in children. The findings were integrated into the game design process and the subsequent clinical evaluation.

### Design Foundations

To ensure CliniPup could achieve the intended outcomes, the Scientific Foundations established in the first phase guided the choice of game mechanics, design, and technological features. Therefore, the theoretical basis was translated into relevant, implementable game design elements.

#### Game Mechanics

Game mechanics (GMs) are rules or methods that define the interactions and flow of a game session. They describe interactions, game conditions, and triggers in an abstract manner. In 2015, Arnab proposed a model for translating learning objectives into learning mechanics (LM) and mapping these to relevant GMs [48]. This so-called LM-GM model guides developers in the development of more effective, pedagogy-driven serious games, as it ensures that game mechanics are selected based on their ability to contribute toward the intended outcomes [48]. The LM-GM model was utilized to map GMs to the learning objectives identified in the Scientific Foundations stage. For example, one such learning objective was to recall the sequence of events for the upcoming procedure (knowing what to expect and do). This involves Blooms’ Ordered Thinking Skills “understanding” and “retention.” Based on the LM-GM model, several LMs address these thinking skills: “exploration,” “repetition,” and “planning” [48]. Each of these LMs was, in turn, mapped to one or more GMs such as “story,” “cascading information,” and “strategy/planning” [48]. As such, the scientific foundations established in Stage 1 were explicitly and systematically translated into the game construct. The implementation of selected GMs in the resulting serious game was then documented to retrospectively assess the translation to the game development stage and ensure that the selected GMs were indeed applied.

#### Design Requirements

Based on findings from the biomedical literature and through interaction with key stakeholders (nurses, pediatricians, parents, etc), a time-motion exercise was carried out to map anxiety and pain throughout a child’s (and their parents) surgical journey. Additionally, when and where (which setting) each of the outcome determinants was most relevant was identified. In turn, this information helped to define various aspects of the serious game such as which characters (stakeholders) should be included, the relevant settings to incorporate, key time points to address, and the most applicable visuals and content elements.

### Game Development and Description

The game development stage represented the creative translation of all evidence collected in the preceding stages toward a fun and engaging educational tool. The learning objectives, GMs, and design requirements were, in particular, used as a framework to ensure the resulting serious game was evidence based. An iterative, stepwise process was carried out, in which a storyboard was first created followed by the development of visuals, graphics, and a user interface. Utilizing a participatory design approach, feedback of clinicians was sought to refine the serious game. The visuals and user interface were generated using Articulate Storyline, a computer program typically used in the development of electronic learning.

## Results

### Scientific Foundations

#### Target Audience

Based on review of the literature, the target audience was defined as children aged 6-10 years with a high unmet medical need at the individual and population level. Although other age segments also showed unmet needs, children aged 6-10 years were selected due to the substantial physical, psychological, social, and economic burdens associated with inadequate preparation and management [5,8,19,28,29]. Moreover, management for children in this segment was considered especially challenging because of several unique characteristics: need for support and information, need for control, capacity to understand age-appropriate information, lack of communication and coping skills, and limited understanding of the surgical purpose and journey [8,19,30,41,49]. In addition, this segment undergoes a robust amount of ambulatory procedures each year [1,3].

Due to the relationship between parental factors (knowledge, skills, behavior, etc) and children’s pain and anxiety, parents are also an important target audience for a nonpharmacological intervention [8,9,16,28]. Therefore, parental unmet needs must be addressed simultaneously. Parental needs are mainly related to coping and communication skills, support, and information on the surgery process and journey [8,19,25,29].

#### Outcome Objectives

There is a particular need to educate children and their parents about the concepts of pain and anxiety, allow them to explore the surgical pathway, and change their behavior with respect to communication and coping [8,19]. These needs are intended to indirectly, or directly, address the overarching goals, which are to reduce child anxiety and pain in the perioperative setting.

#### Theoretical Basis

The literature search, described in the Methods, resulted in the selection of nine articles for inclusion in the study [8,17,18,25,29,30,49-51].

After review, it was clear that pain and anxiety have sensory, emotional, cognitive, and behavioral components that are interrelated with environmental, developmental, sociocultural, and contextual factors [8,12,52]. A number of determinants of pain and anxiety were considered fixed (eg, risk factors) or nonmodifiable and could not be addressed with an intervention (eg, child demographics, parent demographics, and type of procedure) [17,30]. Seven key modifiable determinants were identified: medical fears, current state, sense of control, ability to communicate, understanding (information/knowledge), focus on pain, and parental fears. These seven determinants were generated through categorization of approximately 100 relevant underlying parameters, which were directly defined in the literature. The determinants were also closely aligned with clinical experience suggesting that the following psychological factors are known to influence pain and therefore should be addressed by nonpharmacological interventions: vigilance to pain, avoidance, anger, involvement of the patient, making sense of the pain, and consistency [53]. In turn, the learning objectives were defined for each determinant, and a theoretical mechanism was defined as information, motivation, or behavioral skills, consistent with the IMB model (Multimedia Appendix 1).

As the focus of the development was on a serious game directed at children, it was determined to be infeasible (and impractical) to address the parental learning objectives alongside the child’s learning objectives in a single serious game. However, the parental learning objectives remain critical factors that should be addressed through a complementary or supplementary mechanism or medium.

The learning objectives were further categorized together to yield 11 identified objectives (Table 1).

#### Tool Evaluation

Common approaches in the field to evaluate interventions leveraged structured clinical trials, and therefore, the researchers determined that the optimal approach to evaluate CliniPup was with a randomized controlled clinical trial [35,37,38]. With this in mind, a positive first step is to validate CliniPup in a pilot study before considering a larger, pivotal trial. Through this process, key validated outcome measures were identified for pediatric anxiety and pain and parental anxiety. These measures were examined using the modified Yale preoperative anxiety scale, the Wong-Baker Faces Pain Rating Scale (WBFPRS), and the state-trait anxiety inventory [10,54,55]. In addition to the clinical measures, an evaluation of user satisfaction and experience could also be performed in a pilot trial. Data on these aspects could be collected using structured questionnaires and Likert scales.

### Design Foundations

Screenshots of CliniPup are shown in Figures 2-15. LMs and GMs were selected based on Bloom’s ordered thinking skills that were aligned with the learning objectives (Multimedia Appendix 1), consistent with the LM-GM model (Table 1) [48]. Further, the implementation of the GMs at the development stage is also presented in Table 1 for complete transparency.

#### Design Requirements

Based on the biomedical literature review and interviews with clinicians, it was established that the characters portrayed in the serious game needed to be realistic, yet age-appropriate, especially when visualizing medical professionals [8,41]. Parents should be visualized as kind, gentle, and comforting [8,19,44]. In addition, the literature indicated that the game’s protagonist should be authoritative; likable; relatable; and neutral in gender, age, and ethnicity [8]. Aligned with these points, the use of a nonhuman protagonist, such as an animal, was considered. Further research indicated that animals may offer a positive, comforting role model for children, and therefore, a dog, called CliniPup, was developed to represent the serious game’s protagonist [56,57].

The design requirements for the serious game’s settings were informed mainly by the ethnographic research performed (eg, time-motion exercise and interaction with medical stakeholders). This research suggested that the settings should be linked to the surgical journey and therefore cover the following environments: at home before the surgery, at the hospital before the surgery, at the hospital in the operating room, at the hospital after the surgery, and at home after the surgery [8,19]. The relevance of each of the seven determinants to the different settings was mapped using the time-motion exercise.

Due to the unique pedagogical requirements (eg, short attention span and limited language capabilities) of children aged 6-10 years, the serious game needed to be highly engaging, interesting, and fun [19]. In particular, age-appropriate visuals and language were recommended for educational tools used in health care settings targeted at children in order to enhance engagement [8,17,19]. This suggested that the use of dynamic animations, sounds, voice-overs, and limited text would be optimal. These choices are aligned with the Gestalt theory, which recommends that designers should limit cognitive efforts for optimal communication, particularly in health care settings [58,59]. Additionally, the use of multimedia techniques increases the likelihood of addressing heterogeneous learning styles, facilitating education in a diverse target audience [8,59,60].

#### Tool Evaluation: Implication on Design

With the pilot study design, we expected children to play the serious game CliniPup in their home setting and believed that all outcome data would be collected outside of the game itself. Therefore, minimal design requirements were necessary to accommodate the trial. At-home play was facilitated by designing the serious game to be accessible online and functional in both mobile and desktop environments. In addition, the serious game was linked to a database to allow for the collection and storage of demographic and user interaction data (eg, number of times played).

#### Game Development and Description

**Figure 2:**
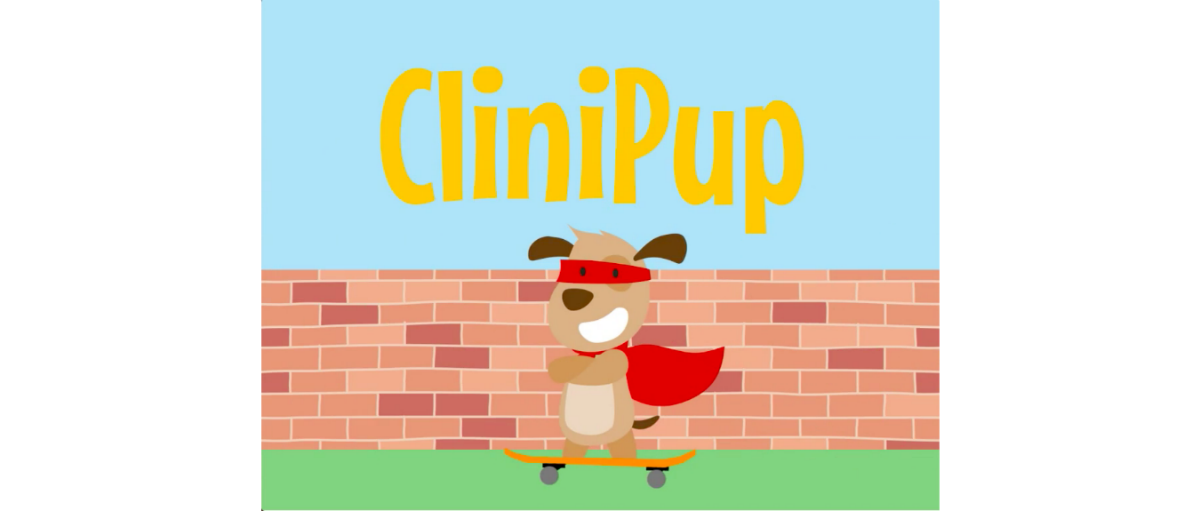
CliniPup introduction and visuals.

**Figure 3:**
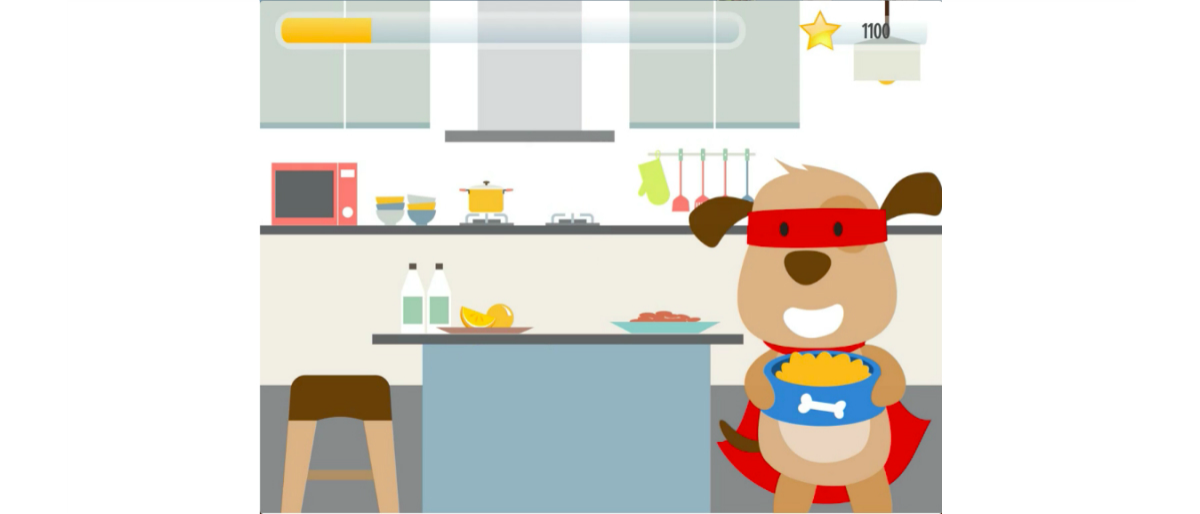
CliniPup narrative visuals: At home before surgery.

**Figure 4:**
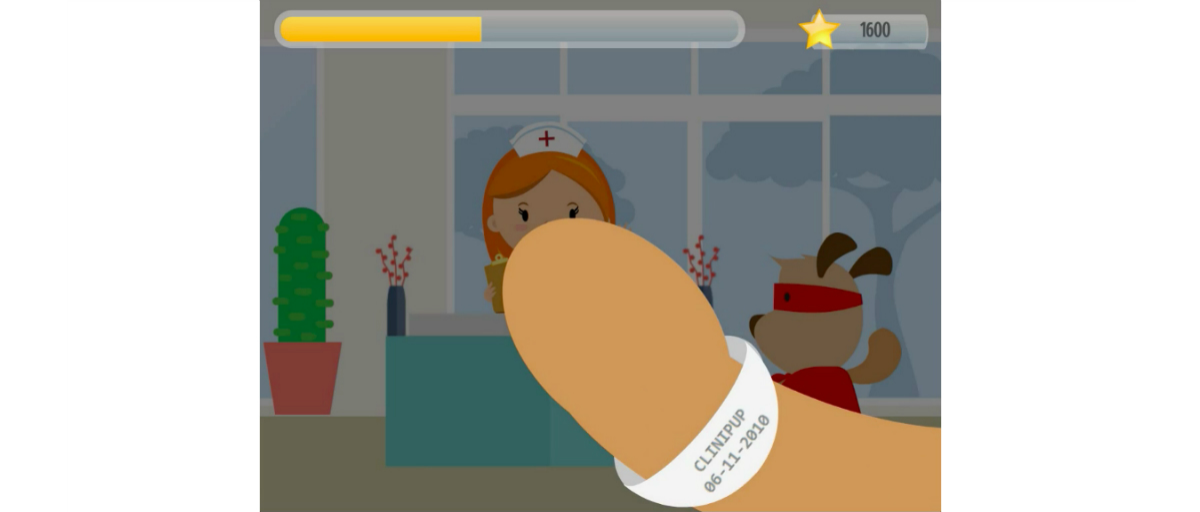
CliniPup narrative visuals: At the hospital before surgery.

**Figure 5:**
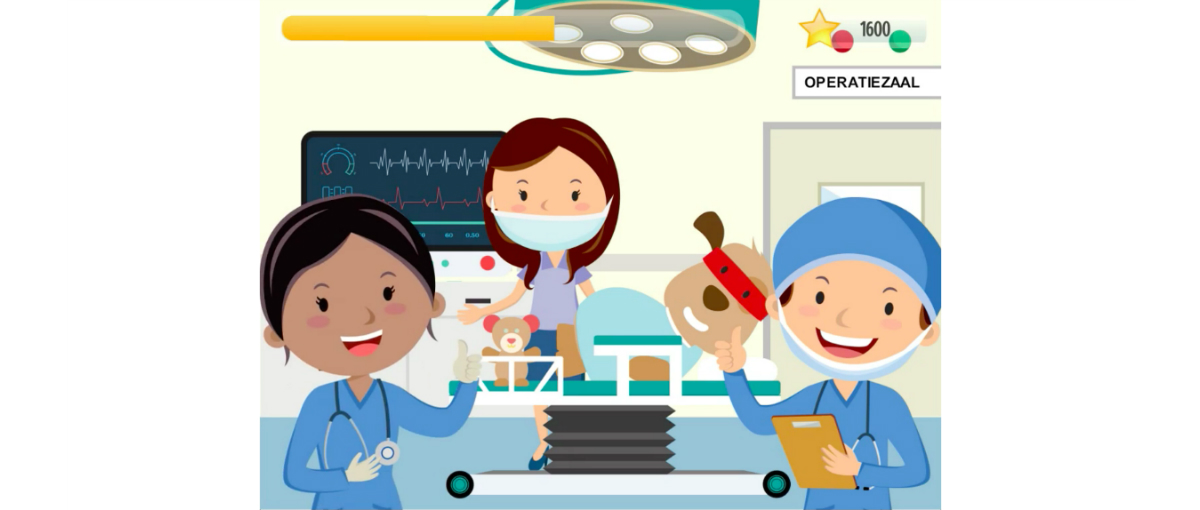
CliniPup narrative visuals: In the operating room.

**Figure 6:**
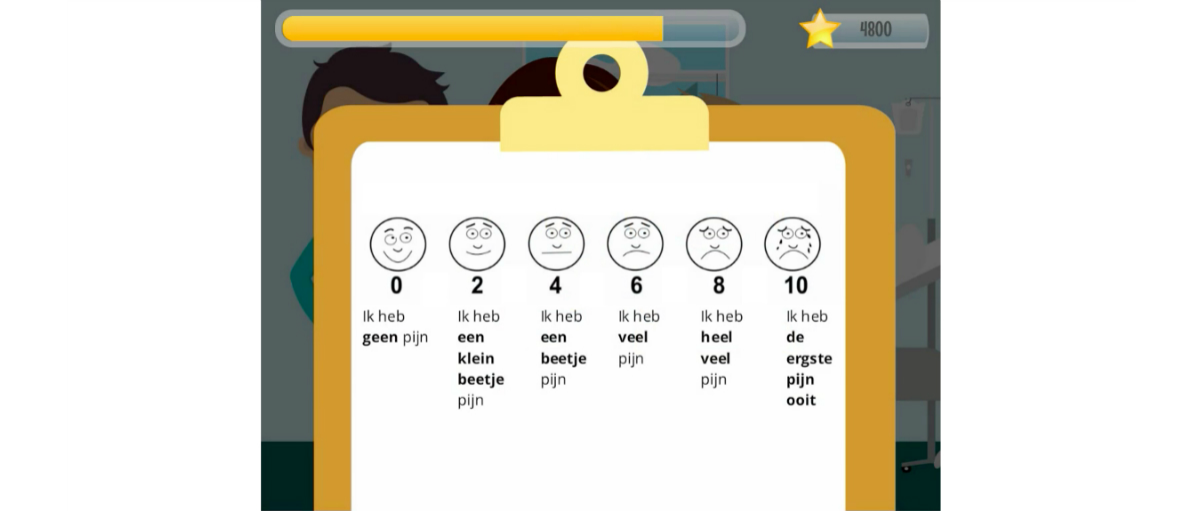
CliniPup narrative visuals: In the hospital after surgery.

**Figure 7:**
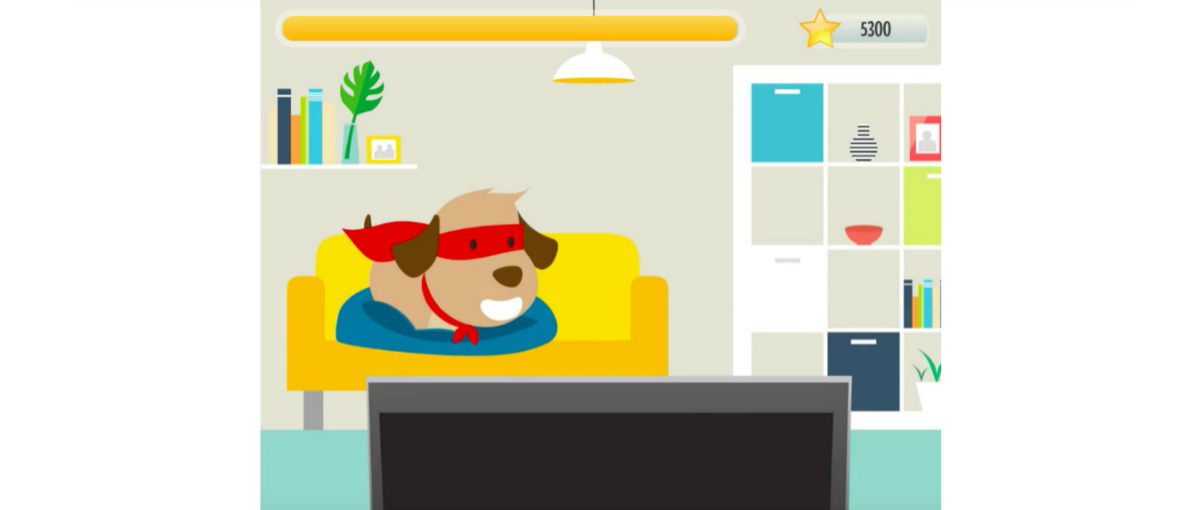
CliniPup narrative visuals: At home after surgery.

CliniPup was developed based on evidence generated from the Scientific Foundations and Design Foundations. A general narrative that explored the ambulatory surgical journey was presented, and a user interface was created to realize the narrative, as described in the Design Foundations (Figures 3-7). The use of this narrative was intended to directly address learning objectives 4, 5, and 6 and to indirectly address all other learning objectives listed in Table 1. The user interface was 2D and cartoon-like, aligned with target audience expectations. The protagonist, CliniPup, was visualized as confident, fun, and authoritative (Figure 2).

**Table 1:** Learning objectives, Bloom’s ordered thinking skills, learning mechanics, game mechanics, and game implementation for the serious game CliniPup.

#	Learning objective	Thinking skill	Learning mechanic	Game mechanic	Game implementation
1	Describe hospital environment, identify types of medical staff involved and their role	Retention, Understanding, Applying	Observation, Exploration, Question and answer	Tutorial, Question and answer, Feedback	Hospital environment and medical staff described during narrative (Figure 5) Mini-game exploring objects and people in the operating room (Figures 9-11)
2	Explain relevant age-appropriate medical terms	Retention, Understanding, Applying	Tutorial, Observation, Identification, Question and answer	Tutorial, Question and answer, Feedback, Realism	Hospital environment and medical staff described during narrative (Figure 5) Use of age-appropriate terminology (eg, “sleep doctor”; Figure 10) Mini-game allowing exploration of hospital environment and answering questions related to the hospital environment (Figures 9-11)
3	Distinguish facts and myths related to day surgery (eg, “may not wake up”)	Understanding	Tutorial, Exploration	Tutorial, Story	Linear sequence of events consistent with events of the day of surgery described during the narrative (Figures 3-7) Common myths addressed directly during the narrative
4	Recall the sequence of events for the upcoming procedure	Retention, Understanding, Applying	Exploration, Repetition, Strategy/planning, Question and answer	Story, Tutorial, Question and answer, Feedback	Linear sequence of events consistent with events of the day of surgery described during the narrative (Figures 3-7) Mini-game about the sequence of events on the day of surgery (Figure 12)
5	Plan the at-home steps to prepare for the surgery	Understanding, Applying	Tutorial, Question and answer	Tutorial, Question and answer, Feedback	At-home sequence (Figure 3) Mini-game about the sequence of events at home (Figure 12) Mini-game about control at home (Figure 14)
6	Describe how it may feel on the day of surgery (what, when, why)	Understanding, Applying, Evaluating	Tutorial, Exploring, Question and answer, Action/task	Tutorial, Question and answer, Capture/elimination, Rewards/penalties	Linear sequence of events consistent with events of the day of surgery described during the narrative (Figures 3-7) Anxiety monsters mini-game at various points of anxiety (Figure 8) In-game score (Figure 15)
7	Explain what anxiety and pain are (manifestation, purpose, transient nature)	Understanding, Applying	Tutorial, Question and answer	Tutorial, Question and answer	Age-appropriate feeling described during the narrative (butterflies, lump in throat, etc) Choosing where anxiety is experienced somatically Anxiety monsters are transient (not constant)
8	Recognize when they are experiencing anxiety and pain (telltale signs)	Understanding, Analyzing, Evaluating	Tutorial, Action/task	Tutorial, Feedback, Capture/elimination, Rewards/penalties	Age-appropriate explanation of anxiety and pain during narrative Anxiety monsters mini-game at various points of anxiety (Figure 8) Demonstration of WBFPRS^a^ (Figure 6) In-game score (Figure 15)
9	Demonstrate confidence to discuss with parents, caregivers, or medical staff	Understanding, Applying	Tutorial, Question and answer	Question and answer	Communication with HCPs^b^ and parents reinforced as positive Mini-game about options related to control on day of surgery (Figure 14)
10	Describe and label feelings of pain or anxiety	Understanding, Analyzing	Tutorial, Question and answer	Question and answer, Feedback	Age-appropriate explanation of anxiety and pain Demonstration of WBFPRS in the narrative (Figure 6) Mini-game with WBFPRS (Figure 13)
11	Use coping skills for dealing with anxiety and pain	Understanding, Applying, Evaluating	Tutorial, Questions and answer, Assessment	Tutorial, Question and answer, Feedback, Action points	Dealing with anxiety explored during the narrative (distraction, communicating, choosing “Clini-buddy,” etc) Mini-game about options related to control on day of surgery (Figure 14)

^a^WBFPRS: Wong-Baker Faces Pain Rating Scale.

^b^HCP: health care practitioner.

**Figure 8:**
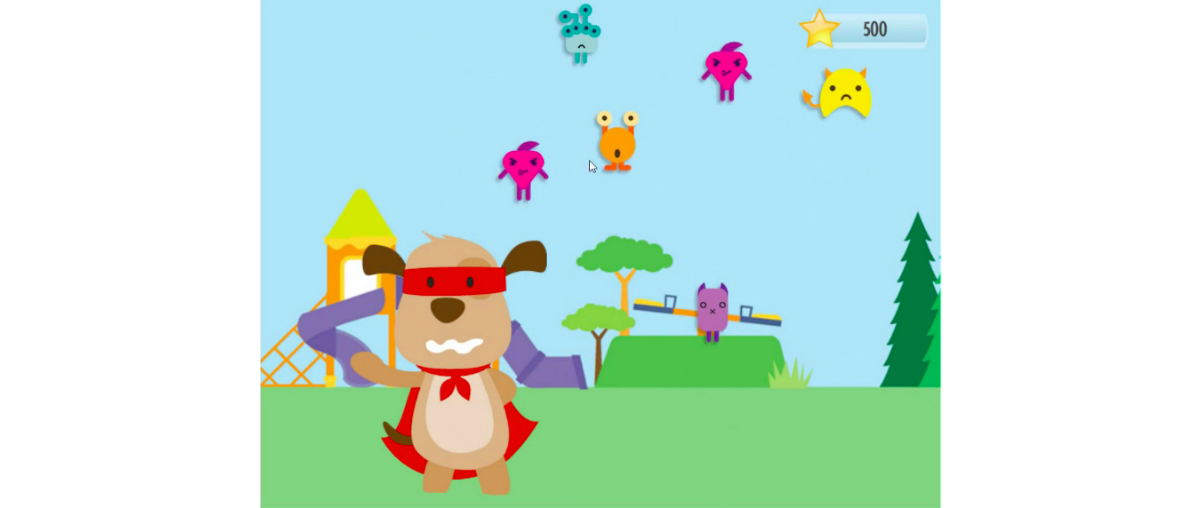
CliniPup anxiety monster mini-game.

**Figure 9:**
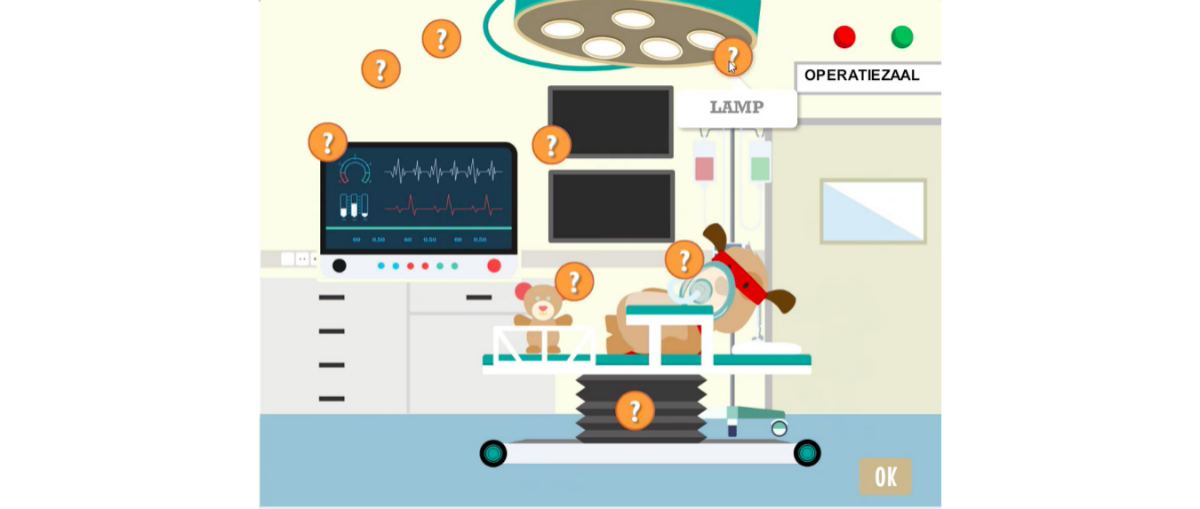
CliniPup quizzes and challenges: Operating room and object names.

**Figure 10:**
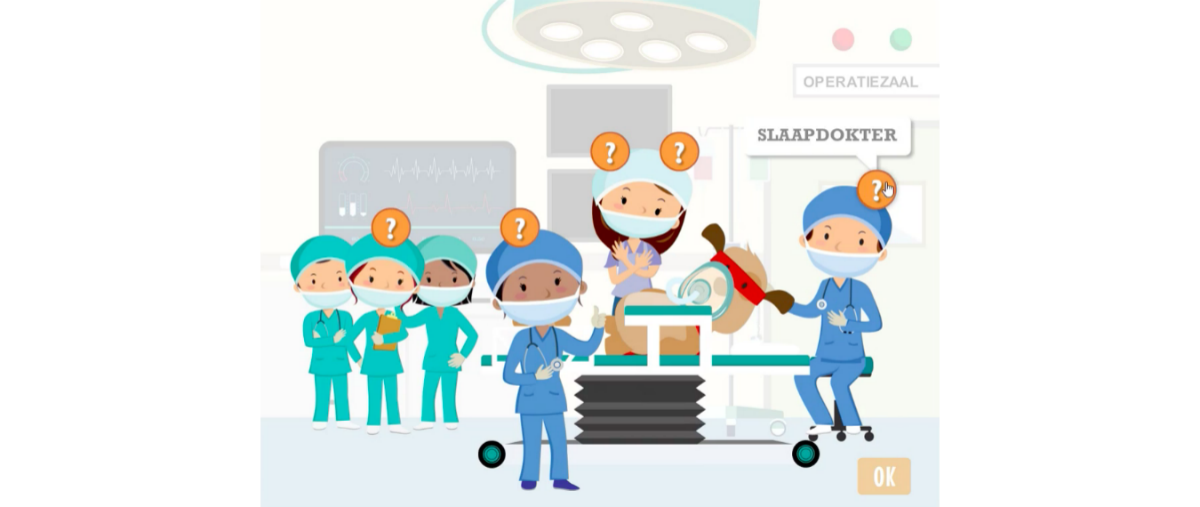
CliniPup quizzes and challenges: People in the operating room.

**Figure 11:**
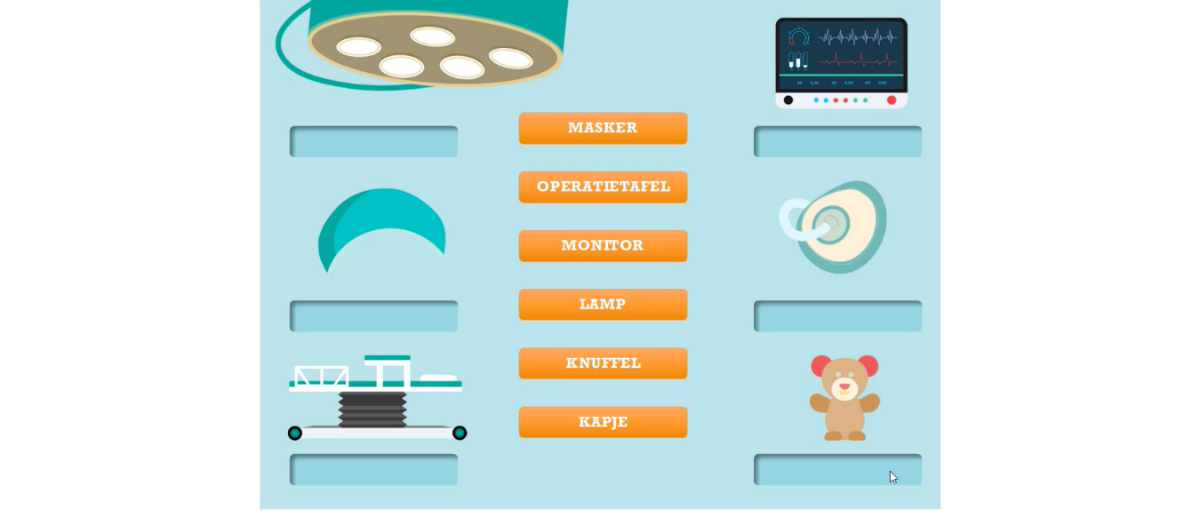
CliniPup quizzes and challenges: Operating room and matching.

**Figure 12:**
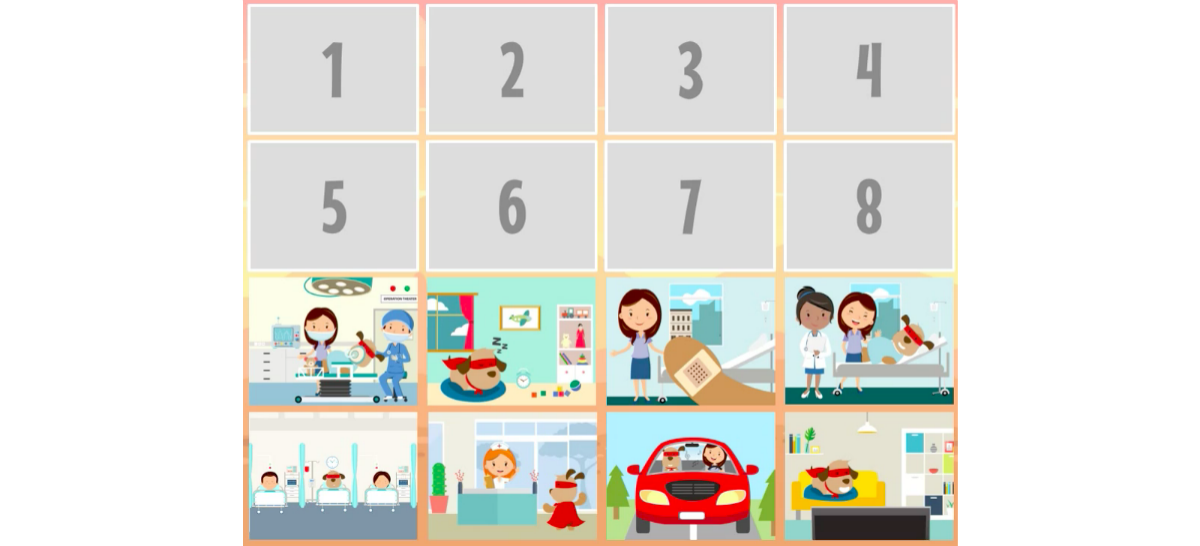
CliniPup quizzes and challenges: Sequence of events throughout the day of surgery.

**Figure 13:**
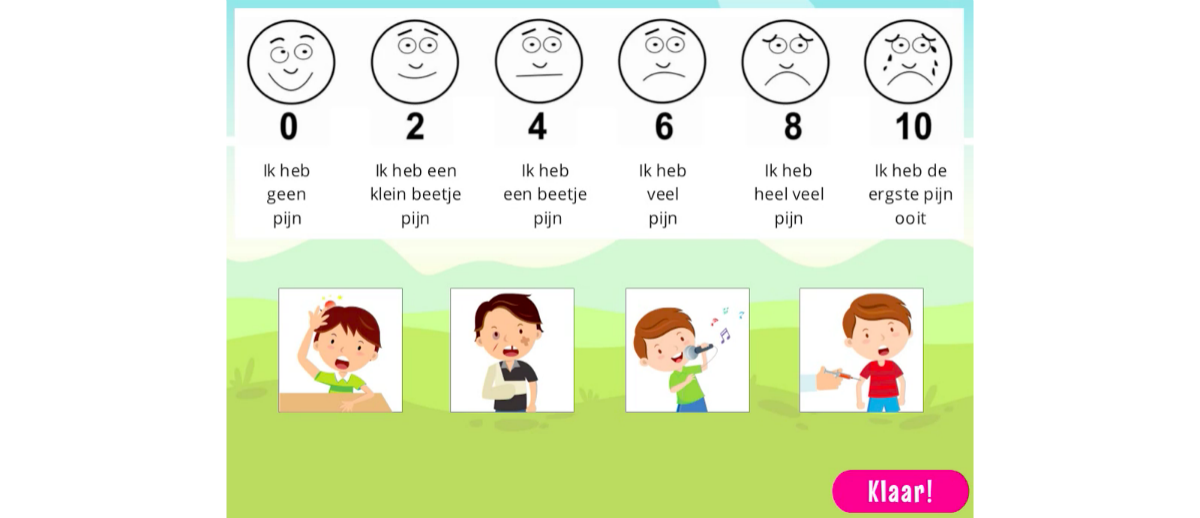
CliniPup quizzes and challenges: Wong-Baker Faces Pain Rating Scale and matching.

**Figure 14:**
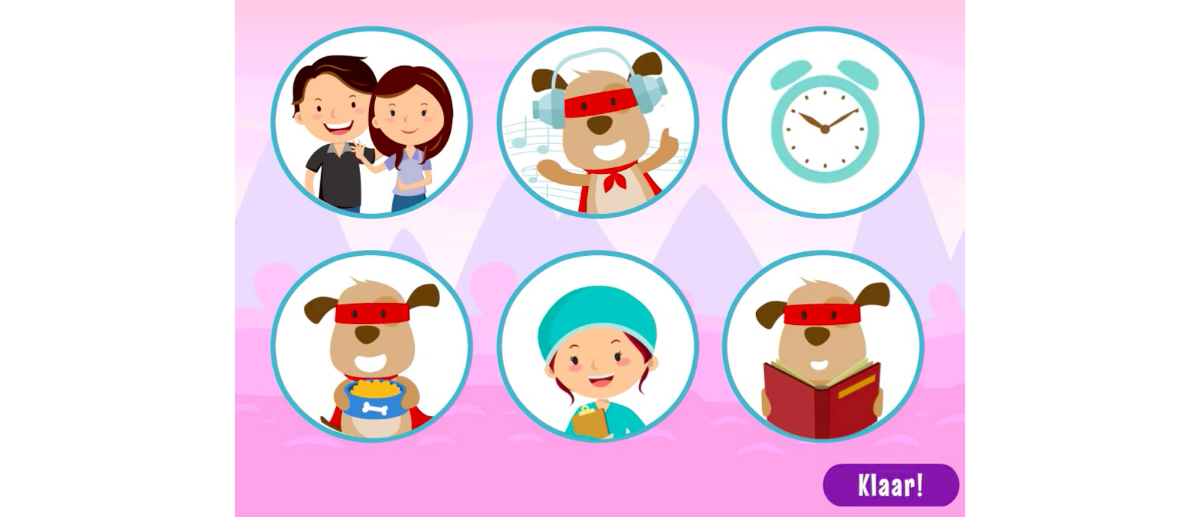
CliniPup quizzes and challenges: Options for exercising control throughout the day of surgery.

**Figure 15:**
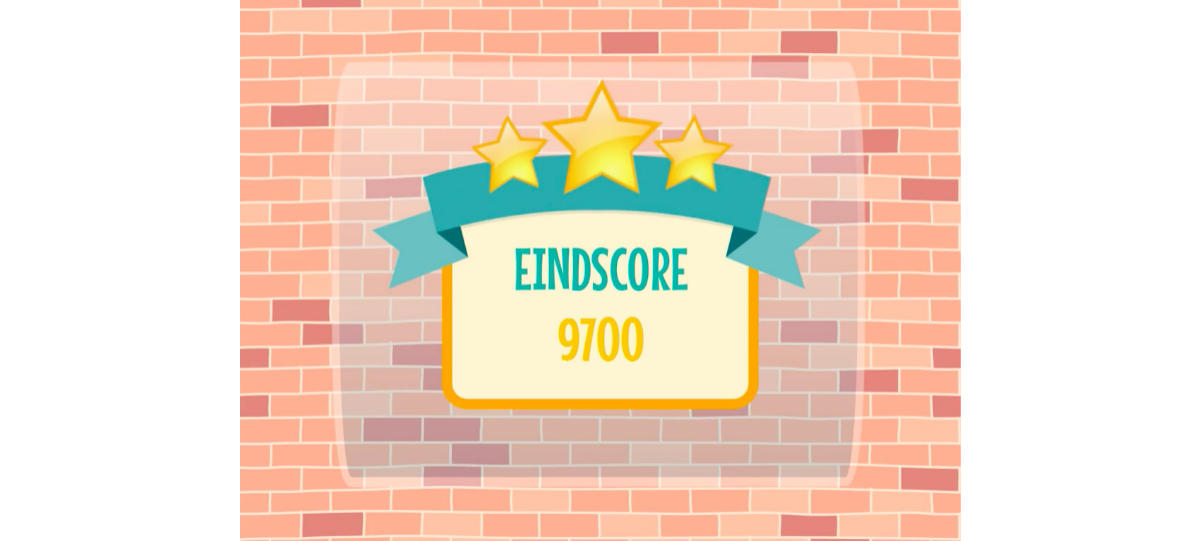
CliniPup final score screen.

Consistent with the design requirements, minimal text was presented and voice-overs and sounds were implemented throughout. In particular, the narrative followed the five key settings of the surgical journey defined in the Design Requirements (Figures 3-7). The tool was accessible online and could be played on a personal computer or tablet.

“Anxiety monsters” would appear at key moments (defined by the time-motion exercise), in which users needed to help the protagonists chase them away (by clicking on the monsters; Figure 2). The purpose of this mini-game was to achieve learning objectives 8, 9, 10, and 11 (Table 1), and it served the purpose of collecting points for the user, which represented the overarching in-game objective (Figure 15).

At the end of the narrative (end of the surgical journey), users were presented with a number of challenges (questions/answers, puzzles, etc) to reinforce the key messages associated with the learning objectives (Table 1). For example, users review the objects (Figure 9) and the people that are present in the surgical room (Figure 10). Moreover, users are asked about the objects in the surgical room to address learning objectives 1 and 2 (Figure 11). In addition, users are requested to identify the correct sequence of events on the day of surgery in accordance with learning objective 4 (Figure 12). Users are also tasked with choosing the correct score on the WBFPRS with various examples to address learning objectives 8 and 9 (Figure 13). Additionally, users must choose actions that are aligned with control to address learning objective 11. In each mini-game, users receive points for completing the challenges correctly (Figure 15).

Based on the number of points collected, users would enter a final “anxiety-monster” mini-game (Figure 8) after which their final score would be presented (Figure 15).

The estimated gameplay time was 20 minutes. Additional information related to the game description is documented in Multimedia Appendix 2.

## Discussion

### Principal Findings

This paper offers a systematic description of the ideation, design, and development of CliniPup, a serious game aimed at reducing perioperative anxiety and pain in children. Aligned with the requirements of serious game stakeholders, the SERES Framework for serious game development [47] was followed to guide the process. Using this framework and within the context of the pediatric perioperative setting, the target audience, outcome objectives, and theoretical bases were assessed and defined within scientific foundations. This evidence was used to select the game mechanics and describe the design requirements (Design Foundations). Subsequently, the Scientific and Design Foundations were explicitly translated into the serious game, CliniPup (game development and design). This high-level process is necessary to ensure that a serious game is based on strong evidence and sound change theories, which is consistent with the best practices in serious game research and development [47]. Moreover, a participatory design approach was used to ensure that the opinions and perspectives of key clinical stakeholders (surgeons, pediatricians, nurses, etc) were integrated throughout the process.

The result of this approach was the serious game CliniPup, which was developed to prepare children for surgery by delivering age-appropriate information, motivation, and behavioral skills in an engaging and fun manner. As such, CliniPup has the potential to address the limitations of existing nonpharmacological tools—cost, time, and accessibility—and differentiate them from other digital interventions, which are focused purely on distraction rather than preparation and empowerment [8,9,19,33,40].

### Limitations

One limitation of the present study was that there was minimal participation from end users in the preliminary phases of design and development. The consequences of this are that children may not find CliniPup informative, believable, or fun. However, the perspectives and expertise of clinical experts in the field of pediatric surgery were consistently leveraged and a comprehensive evaluation with end users is planned for the next step. Moreover, real-world evidence was collected to supplement scientific and clinical evidence. This real-world evidence was generated through ethnographic research such as several time-motion exercises in which research nurses were shadowed on the day of surgery to ensure that a solid understanding of children and parent perspectives and experiences was collected. In addition, all learning objectives defined were not explicitly addressed with the current version of CliniPup, as it was deemed infeasible to simultaneously focus on parents’ and children’s learning objectives in a single serious game. Parental learning objectives could be implicitly addressed if parents play CliniPup with their children, but this is not expected to be an optimal mechanism. Nevertheless, the parental learning objectives could be achieved by the development of a supplementary module or educational tool focused on the parent, which uses language, visuals, content, and other tools aligned with their educational needs.

### Future Research

The next step in the development process is to evaluate the current version of CliniPup with the target audience and collect their feedback on a range of factors such as usability, satisfaction, and learning experience. Data will also be collected on key clinical endpoints to develop a preliminary understanding of CliniPup’s effect in the target audience. These factors will be evaluated in a pilot trial with children undergoing ambulatory surgery and their parents. The pilot study will provide feedback on the experience of the target audience; this feedback will be leveraged to refine and improve CliniPup. Beyond this, a subsequent step is the development of a complementary tool targeted toward parents, which will ensure that all learning objectives are addressed.

### Conclusions

This is the first paper to provide a comprehensive description of the ideation and development of a serious game aimed at reducing perioperative anxiety and pain in children. The development of CliniPup followed the SERES Framework for serious game development and therefore was based on strong scientific evidence and sound change theories. A key component of this approach was to generate a clear understanding of the unmet need, formulate learning objectives, and develop a serious game that can realize these objectives. As such, CliniPup can address the limitations of current nonpharmacological interventions and is different from other digital interventions such as tablet-based distraction. The usability, satisfaction, and initial clinical findings associated with CliniPup were subsequently evaluated in a pilot trial. Findings from this evaluation will inform the next steps in the serious game development process, reflecting the participatory and iterative design approach that is central to the SERES Framework for serious game development.

## Appendix

Multimedia Appendix 1 Matrix of determinants, mechanisms, and learning objectives to reduce perioperative anxiety and pain in children.

Multimedia Appendix 2 Description of game design elements implemented in the serious game CliniPup.

## Abbreviations

GM: game mechanic
HCP: health care practitioner
IMB: Information-Motivation-Behavioral skills
LM: learning mechanic
WBFPRS: Wong-Baker Faces Pain Rating Scale
