# Supplementary material for: Development of CliniPup, a Serious Game Aimed at Reducing Perioperative Anxiety and Pain in Children: Mixed Methods Study
Source: JMIR Serious Games. 2019 Jun 1;7(2):e12429. doi: 10.2196/12429 (PMC6592492; doi:10.2196/12429)
Supplement: Multimedia Appendix 1 [file games_v7i2e12429_app1.docx]

## Appendix A: List of determinants, mechanisms, and learning objectives to reduce perioperative anxiety and pain in children.

| **Determinant** | **Mechanism** | **Learning Objectives** | | | **Key sources** |
| --- | --- | --- | --- | --- | --- |
|  |  | **Information** | **Motivation** | **Behavioral skills** |  |
| Medical fears | Reduce fears | 1. Identify types of medical staff involved in procedure and their roles  2. Describe hospital environment  3. Explain age appropriate medical terms | 4. Distinguish facts from myths related to surgery | NA | [8, 30] |
| Current state | Alleviate | NA | 5. Recall how s/he will feel on day of the surgery  6. Explain why s/he will feel this way  7. Recall that the feelings are temporary | NA | [8, 17] |
| Sense of control | Increase control  Develop | 10. Recall the sequence of events for the upcoming procedure | NA | 8. Use coping skills for dealing with anxiety  9. Use coping skills for dealing with pain  11. Plan the at-home steps to prepare for the surgery | [17] |
| Ability to communicate | Develop communication skills | 13. Actively use age-appropriate terminology | 12. Demonstrate confidence to discuss any questions/worries with parents, caregivers, or medical staff | 14. Describe pain or anxiety and formulate worries  15. Differentiate between levels of pain severity and demonstrate ability to use visual pain scale | [30] |
| Understanding | Increase understanding | 16. Explain what anxiety is  17. Explain the purpose of pain and its transient nature | NA | NA | [17, 18] |
| Focus on pain | Reduce focus on pain | 18. Anticipate when s/he will experience anxiety/pain  19. Recognize when s/he actually experiences anxiety/pain | NA | 20. Distract themselves from pain | [25] |
| Parental fears | Reduce parental fears | 21. Recall the sequence of events for his/her child’s procedure  22. Describe his/her role in preparing his/her child for surgery  23. Describe his/her role in managing his/her child ‘s pain at home | 24. Distinguish facts from myths related to surgery  25. Recognize the impact of his/her child’s anxiety | 26. Communicate appropriately with his/her child about pain and anxiety | [29] |
