# Supplementary material for: Development of CliniPup, a Serious Game Aimed at Reducing Perioperative Anxiety and Pain in Children: Mixed Methods Study
Source: JMIR Serious Games. 2019 Jun 1;7(2):e12429. doi: 10.2196/12429 (PMC6592492; doi:10.2196/12429)
Supplement: Multimedia Appendix 2 [file games_v7i2e12429_app2.docx]

## Appendix B: Description of game design elements implemented in the SGH, CliniPup

| **Game design categories** | **Game design elements** | **Description** |
| --- | --- | --- |
| Genre | Genre | Narrative-based simulation; question/answer; puzzles |
| Game platform | Game platform | PC or tablet PC |
| Game engine | Game engine | NA |
| Data | Protection | Data collected in secure database |
|  | Collection | Demographic information, user interactions |
| Game objectives | Game objectives | Obtain high score through:(a) Clicking anxiety monsters(b) Completing challenges correctly(c) Collecting points in mini-games |
| Rules | Rules | Minimal:Linear narrativeTime limitations of mini-gamesSelection of limited options in challenges |
| Content | Characters | CliniPupParent (Mom)CliniciansAnxiety monsters |
|  | Settings | 1. At home before the surgery2. At the hospital before the surgery3. At the hospital in the operating room4. At the hospital after the surgery 5. At home after the surgery |
|  | Delivery mechanism | Voiceover & user self-discovery via interactivity |
| Narrative | Storyline | 1. At home before the surgery2. At the hospital before the surgery3. At the hospital in the operating room4. At the hospital after the surgery5. At home after the surgery 6. Post-game evaluation of knowledge transfer  7. Mini-games |
| Interactivity | Interactions | Clicking anxiety-monsters  - Self-discovery of content - Completing challenges |
| Feedback | Feedback | Discrete feedback related to the challenges  - Positive voice-over feedback - Feedback aligned with learning objectives |
| Training/support required | Training/support required | Minimal. Game is designed for children to play independently but parents may play with them. |
| Development tool | Development tool | Articulate 360 |
